# Parasite genetic diversity reflects continued residual malaria transmission in Vhembe District, a hotspot in the Limpopo Province of South Africa

**DOI:** 10.1186/s12936-021-03635-z

**Published:** 2021-02-16

**Authors:** Hazel B. Gwarinda, Sofonias K. Tessema, Jaishree Raman, Bryan Greenhouse, Lyn-Marié Birkholtz

**Affiliations:** 1 Malaria Parasite Molecular Laboratory, Department of Biochemistry, Genetics and Microbiology, Institute for Sustainable Malaria Control, University of Pretoria, Private Bag X20, Hatfield, 0028 Pretoria, South Africa; 2 Division of HIV, Infectious Diseases, and Global Medicine, Department of Medicine, University of California San Francisco, San Francisco, CA USA; 3 Centre for Emerging Zoonotic and Parasitic Diseases, National Institute for Communicable Diseases, a Division of the National Health Laboratory Service, Gauteng, South Africa; 4 Wits Research Institute for Malaria, Faculty of Health Sciences,, University of Witwatersrand, Johannesburg, Gauteng South Africa

**Keywords:** *Plasmodium falciparum*, Genetic diversity, Microsatellites, Multiplicity of infection, Residual transmission, South Africa, Vhembe District, Limpopo

## Abstract

**Background:**

South Africa aims to eliminate malaria transmission by 2023. However, despite sustained vector control efforts and case management interventions, the Vhembe District remains a malaria transmission hotspot. To better understand *Plasmodium falciparum* transmission dynamics in the area, this study characterized the genetic diversity of parasites circulating within the Vhembe District.

**Methods:**

A total of 1153 falciparum-positive rapid diagnostic tests (RDTs) were randomly collected from seven clinics within the district, over three consecutive years (2016, 2017 and 2018) during the wet and dry malaria transmission seasons. Using 26 neutral microsatellite markers, differences in genetic diversity were described using a multiparameter scale of multiplicity of infection (MOI), inbreeding metric (Fws), number of unique alleles (A), expected heterozygosity (*He*), multilocus linkage disequilibrium (LD) and genetic differentiation, and were associated with temporal and geospatial variances.

**Results:**

A total of 747 (65%) samples were successfully genotyped. Moderate to high genetic diversity (mean *He* = 0.74 ± 0.03) was observed in the parasite population. This was ascribed to high allelic richness (mean A = 12.2 ± 1.2). The majority of samples (99%) had unique multi-locus genotypes, indicating high genetic diversity in the sample set. Complex infections were observed in 66% of samples (mean MOI = 2.13 ± 0.04), with 33% of infections showing high within-host diversity as described by the Fws metric. Low, but significant LD (standardised index of association, ISA = 0.08, *P < 0.001*) was observed that indicates recombination of distinct clones. Limited impact of temporal (F_ST_ range − 0.00005 to 0.0003) and spatial (F_ST_ = − 0.028 to 0.023) variation on genetic diversity existed during the sampling timeframe and study sites respectively.

**Conclusions:**

Consistent with the Vhembe District’s classification as a ‘high’ transmission setting within South Africa, *P. falciparum* diversity in the area was moderate to high and complex. This study showed that genetic diversity within the parasite population reflects the continued residual transmission observed in the Vhembe District. This data can be used as a reference point for the assessment of the effectiveness of on-going interventions over time, the identification of imported cases and/or outbreaks, as well as monitoring for the potential spread of anti-malarial drug resistance.

## Background

Malaria remains a global health problem, with about 228 million cases reported worldwide in 2018, 93% of which occurred in the World Health Organization (WHO) African Region. As the southern Africa region, excluding high-transmission countries like Mozambique, accounted for < 10% of the 213 million cases reported in the WHO African region, several southern African countries have been earmarked for malaria elimination by 2023 guided by the WHO Global Technical Strategy for Malaria [1]. Unfortunately, like a number of other regions in the world, southern Africa experienced a resurgence in malaria cases and deaths during the 2017/2018 season [2]. This resulted in South Africa reporting more than 30 000 cases, a surge in numbers previously only experienced during the 1999/2000 drug and insecticide resistance outbreak [3–5].

**Fig. 1:**
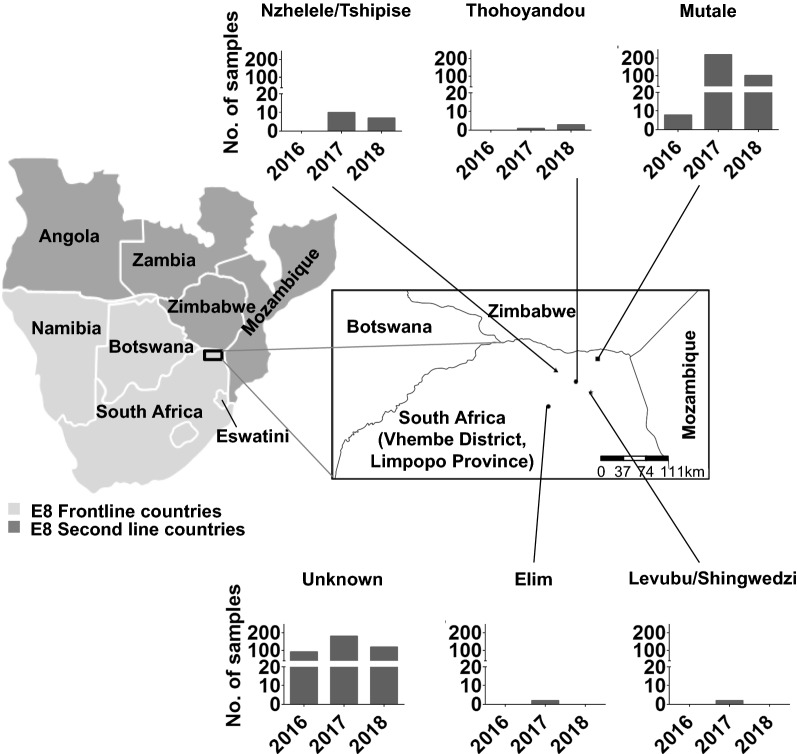
Study area and design. Map of the study site in the Vhembe District, Limpopo Province, South Africa. Bar graphs show the proportion of samples collected from the different source health districts and the years of transmission. The study district shares borders with Zimbabwe, Botswana and Mozambique. South Africa is shown alongside other malaria endemic countries in the E8 region. The “unknown” category represents 45% of the samples that could not be linked to source health districts

*Plasmodium falciparum* is the predominant species which accounts for the majority of cases and fatalities in the South Africa [5], with *Anopheles funestus* and the *Anopheles gambiae* complex the main vector species associated with transmission, which mainly occurs in the hot and rainy season between September and May [5]. While South Africa has made significant progress in the reduction in malaria cases since the 1999/2000 outbreak through the implementation of sustained vector control and case management interventions, progress has stalled [3–9]. The country is characterized with heterogeneous transmission settings in the three remaining endemic provinces: Kwa-Zulu Natal (KZN), Mpumalanga and Limpopo [4, 5]. The Vhembe District in the Limpopo Province with the greatest burden of disease is classified as a moderate transmission area with 3.79 local cases/1000 population at risk in 2018, compared to very few locally transmitted cases (< 0.1 cases/1000 population at risk) reported for the KwaZulu-Natal (KZN) Province [5]. The Vhembe District is situated in the north-eastern border region of the country bordered by Mozambique to the southeast, Zimbabwe to the north and Botswana to the northwest (Fig. 1). This region experiences sustained, seasonal malaria transmission and accounts for 60% of the country’s burden [4]. While importation of cases, mostly from Mozambique and Zimbabwe, has been implicated in on-going transmission in the Vhembe District, the majority (63%) of the cases collected from 1998 to 2017 in the district were classified as from local transmission based on travel histories [4, 10]. Other causative factors for the persistent residual transmission observed in the Vhembe District, despite sustained vector control strategies and public health interventions, include antimalarial drug resistance, insecticide resistance and vector species variance between *An. gambiae* and *An. funestus* [7, 11–15].

The addition of malaria parasite population genetic data to the standard surveillance data collected by malaria control programmes has assisted in understanding malaria transmission dynamics and also allowed for spatio-temporal inferences to be made from e.g. local vs. imported malaria cases [16–24]. These data reflect that typically, parasite genetic complexity decreases with a decline in malaria transmission, which holds true for low transmission settings such as Southeast Asia and Latin America compared to high transmission settings of sub-Saharan Africa based on incidence data. However, the limited data available from microsatellite genotyping of parasites from low transmission settings in sub-Saharan Africa (from the KZN Province in South Africa, Eswatini, and Namibia [9, 25, 26]), using the same approach as in the current study, suggests the opposite. In these study sites, parasites were highly complex and diverse, and while in some of these settings this relatively high diversity was attributed to frequent importations from neighbouring high transmission settings, in other settings this diversity was characterized by local transmissions suggesting that something different is happening to the parasites in the region.

To better understand the malaria parasite-associated factors that contribute to residual malaria transmission in the Vhembe District, microsatellite genotyping was applied to describe the population of *P. falciparum* parasites in South Africa at different spatial and temporal scales. This work identifies the contributing factors associated with sustained residual transmission in the Vhembe District in South Africa as the country works towards malaria elimination.

## Methods

### Study site and study design

Study samples were randomly collected from seven primary health clinics within five known source health districts (Nzhelele/Tshipise, Thohoyandou, Mutale, Elim, and Levubu/Shingwedzi) within the Vhembe District, Limpopo Province, South Africa (Fig. 1). Out of a total of 1892 confirmed cases that occurred during the entire study period and reported only at the 7 study clinics, a total of 1153 (61%) RDT samples from symptomatic patients that tested positive for malaria on *P. falciparum*-specific RDTs (First Response® Malaria Antigen *P. falciparum* card test HRP2, Premier Medical Corporation, India) were randomly collected from the seven different clinics in the wet and dry seasons from January to December of the years 2016, 2017 and 2018. Samples were transported and stored at room temperature in sealed bags with desiccant. Demographic and travel history information on each of the individual patients was obtained from the LMC database. After linking genotyped data with patient information from the LCM database, samples were stratified based on the source of infection health district (Fig. 1) as it was established that patients that acquired their infections from similar source areas did not necessarily visit the same clinics for testing and treatment. Therefore, it was concluded that source health districts would be more informative for geospatial analysis than clinics. 45% (45%) of the samples however, could not be linked to source health districts and were therefore classified as “unknown”. Source of infection could not be established not because information was not available on the LCM database, but because the genotyped samples could not be linked back to patient identity as the only unique identifiers that could link the patient RDT to their information on the database were either missing or illegible on the RDT. The study was started off with blinded patient identities with only knowledge of year of infection and clinic visited by patients from where samples were then collected.

### DNA isolation and quantification

DNA was extracted from the nitrocellulose strip of First Response™ RDTs in accordance with the Worldwide Antimalarial Resistance Network Guidelines [27]. The Saponin-Chelex method [28] was used for all DNA extractions and extracted genomic DNA stored at − 20 °C. Isolates were screened for *P. falciparum* using ultra-sensitive varATS [29] and TARE-2 [29, 30] quantitative PCR (qPCR) as described before. Parasite density was quantified on a random subset of 353 high volume samples from all years. It was not possible to quantify all samples due to a low DNA volume for a proportion of samples. The genotyping threshold was set at a parasite concentration of ≥ 10 parasites/µL of blood.

### Microsatellite genotyping

A total of 1153 samples were genotyped using a previously described 26 microsatellite marker panel protocol [25, 26, 31]. Briefly, the 26 microsatellite loci were amplified using a semi-nested PCR protocol. The primary PCR was performed in 4 groups of multiplex reactions, and 1 µL of the primary amplified product used as a template for the secondary individual PCR for each marker. To determine repeat length sizes, the labelled PCR products were diluted and sized by denaturing capillary electrophoresis on an ABI 3730XL analyser using GeneScan™ 400HD ROX™ Size Standard (Thermo Fisher Scientific). MicroSPAT software (https://github.com/Greenhouse-Lab/MicroSPAT/releases/tag/v2.0.3) was then used to automate identification of true alleles and differentiate real peaks from artefacts of the resulting electropherograms using a classifier algorithm based on the location and size of locus-specific patterns relative to a primary peak as done in studies conducted in Eswatini [25], Namibia [26], the KZN Province of South Africa [9] and China [31] that used similar experimental conditions as those used in his study. Multiple alleles per locus were scored if minor peaks were at least a third of the height of the major peak. Collated genotyping data from all samples was processed with similar microSPAT software settings in which a semi-supervised naïve Bayes classifier was used [25] to avoid variability in allele calling. All samples that met the genotyping threshold were genotyped and had to have a genotyping coverage ≥ 60% (alleles detected on at least 15 or more loci) to be included in the downstream population genetics analysis.

### Characterisation of within-host genetic diversity

The within-host diversity of infections was determined using multiplicity of infection (MOI, or genetically distinct *P. falciparum* clones) and the within-host fixation index (F_WS_). To reduce the probability false positive alleles influencing MOI, the MOI in an individual sample was defined as the second highest number of alleles detected at any of the 26 microsatellite loci genotyped. Since the *P. falciparum* parasite is haploid in the human host, multiple peaks or alleles correspond to an infection with multiple genotypes or strains (a polygenomic or polyclonal infection).

The other measure of the within-host diversity, the F_WS_ index, is a measure of diversity in an individual infection relative to the population level genetic diversity and was determined as previously described [25, 26, 32]. A low F_WS_ indicates high within-host diversity relative to the population thereby suggesting higher chances of outbreeding. F_WS_ was calculated for each sample using the equation: $Fws=1-Hw/Hs$, where *Hw* is the allele frequency of each unique allele found at a particular locus for each individual and *Hs* is the heterozygosity of the local parasite population. Outbreeding is reported as 1-F_WS_ with a 0 value indicative of a perfect clone and therefore low within-host diversity. Previously described thresholds of Fws ≥ 0.95 (1-Fws ≤ 0.05) were used to identify samples containing a single genotype (or “clonal” infections) in spite of additional genotypes that may be present at relatively low proportions; and Fws ≤ 0.70 (1-Fws ≥ 0.30) to describe samples with highly diverse infections respectively [25, 32–34].

### Characterization of population level genetic diversity

Population level genetic diversity was estimated using expected heterozygosity (*He*) which is defined as the probability of randomly drawing a pair of different alleles from the allele pool. Heterozygosity was, therefore, calculated on each locus using the equation: $He=\left[\left(\frac{n}{n-1}\right)*\right(1-\sum {p}_{{i}^{2}}\left)\right]$, where n is the number of genotyped samples and p_i_ is the frequency of the i^th^ allele in the population [25, 26]. Values for *He* range from 0 indicating no diversity, to 1 indicating that all alleles are different and therefore there is maximum diversity. The mean *He* was calculated by taking the average *He* across all loci. The number of haplotypes (unique multilocus genotypes) as well as the unique alleles detected per locus (A - allelic richness), were also determined.

### Linkage disequilibrium

To assess whether alleles from different loci were associated with each other, the multilocus linkage disequilibrium (LD) was determined as previously described [35] using the *Poppr* package in R software [36]. LD was determined for the whole dataset which includes monoclonal and polyclonal infections, with LD for monoclonal infections alone determined as a precaution against the bias that may result from presence of any false dominant haplotypes [16]. The Monte Carlo method was used to test the significance of LD in the complete data set of each population stratified by geographic origin of the infection (source health district). In this study, 10 000 permutations were completed. LD values range from 0 (no loci in LD) to 1 (all loci in LD). Pairwise standardized index of association (ISA) over all loci was assessed to determine whether the observed pattern of LD is due to a single or multiple pairs of loci.

### Geospatial population substructure and genetic differentiation

To determine the influence of geographic origin of infections on genetic diversity, the ANOVA pairwise t-test was used to compare MOI, 1-F_WS_ and *He* between the parasite populations stratified by source health district. Population substructure between the geographic areas was investigated by measuring Wright’s F-statistics (F_ST_), using the *adegenet* package [37] in R. Hendrick’s G_ST_ and Jost’s D, were calculated using the mmod package [38] in R. Genetic differentiation between populations ranges from 0 to 1 representing absence of to complete differentiation, respectively. The Monte Carlo method was used to test the significance of pairwise F_ST_ between source health districts. In this study, 999 permutations were completed. Additionally, discriminant analysis of principal components (DAPC) using the *adegenet* package in R software was used to confirm signatures of population structure [37, 39]. DAPC infers population structure based on whether haplotypes (estimated from multi-locus genotypes generated from all major and minor allele data) clustered into distinct genetic populations. Unlike the traditional principal component analysis (PCA) which identifies linear axes that explain the most variability in all groups together, DAPC seeks to detect the linear axes which explain the most between-group variability in data [37]. K-means clustering was used to detect the number of inferred genetic clusters in the parasite population, and the best number of clusters chosen was that with the lowest associated Bayesian Information Criterion (BIC). To prevent overfitting of clusters, the optimal number of principal components (PC) to be retained was confirmed by cross validation of the DAPC. Data was divided into a training set (90% of data), and a validation set (10% of data), and members of each of the identified clusters were stratified by random sampling to ensure that at least one member of each cluster is represented in both training and validation sets. DAPC was then performed on the training set with variable numbers of PCs retained. The extent to which the analysis was able to accurately predict group memberships of individuals in the validation set was used to identify the optimal number of PCs to be retained. Sampling and DAPC procedures were repeated 1000 times at each level of PC retention, and the optimal number of PCs retained was associated with the lowest root mean square error. The resultant clusters were then plotted in a scatterplot of the first and second linear discriminants of DAPC.

### Characterising pairwise genetic relatedness between infections

To determine the genetic connectivity/relatedness of pairs of infections including all alleles detected in both monoclonal and polyclonal infections of successfully genotyped samples, a modified identity by state (IBS) metric was used [26]. Overall, pairwise IBS was calculated as the average of locus specific estimates under the assumption of independent loci. This metric was calculated using the formula: $IBS= \frac{1}{n}\sum _{i=1}^{n}\frac{Si}{XiYi}$ where *n* is the number of genotyped loci; *S*_*i*_ is the total number of shared alleles at locus *i* between samples *X*; and *Y*_*i*_*X*_*i*_ is the number of alleles in sample *X* at locus *I;* and *Y*_*i*_ is the number of alleles in sample *Y* at locus *i*. A total of 85,078 pairs of infection within the Vhembe District dataset were analysed and highly related infection pairs above the cut-off of IBS ≥ 0.5 [26] identified. Pairwise comparisons of relatedness of parasite pairs were then grouped into two categories depending on whether they occurred between two parasites isolated from individuals who acquired infections in the same (within) source health district or between two parasites from individuals who acquired infections from different (between) source health districts respectively. ANOVA pairwise t-test was used to compare differences in the proportions of highly related infections within and between the groups.

### Influence of temporal variation on genetic diversity

To determine the influence of temporal changes in transmission on genetic diversity, the ANOVA pairwise t-test was used to compare MOI, 1-F_WS_ and *He* between the parasite populations stratified by year of transmission. Finer scale stratification by month of infection was also assessed to determine how MOI changes in the dry and wet transmission seasons and pairwise t-tests were used to compare MOI between the different seasons.

### Assessing the impact of control interventions on the complexity of infections

The impact of the main strategy for control, indoor residual spraying (IRS) on the within-host diversity of *P. falciparum* was also assessed based on the number of unique genotypes (MOI) identified in sprayed vs. unsprayed households. IRS in the Limpopo Province typically takes place at the beginning of each malaria season (wet season between September and May), with the number of households to be sprayed determined by the provincial malaria control programme based on factors such as the number of structures within the malaria endemic area, insecticide availability and resistance data [4].

## Results

A total of 1153 *P. falciparum* RDT positive samples were collected from the Vhembe district, the highest proportion (56%) of which were collected in 2017 and, for those with known source health district data, the majority came from within the Mutale district (Fig. 1). A slightly higher proportion (54 vs. 46%) of females compared to males was sampled during the study with a median age of 23 years, ranging between 19 and 65 years. The median parasite density of a subset of randomly selected quantified samples (n = 313) was 660 parasites/µL of blood, which was above the genotyping threshold of ≥ 10 parasites/µL of blood. All 1153 samples were therefore subjected to microsatellite genotyping.

### Microsatellite genotyping indicates parasite complexity and diversity

**Fig. 2:**
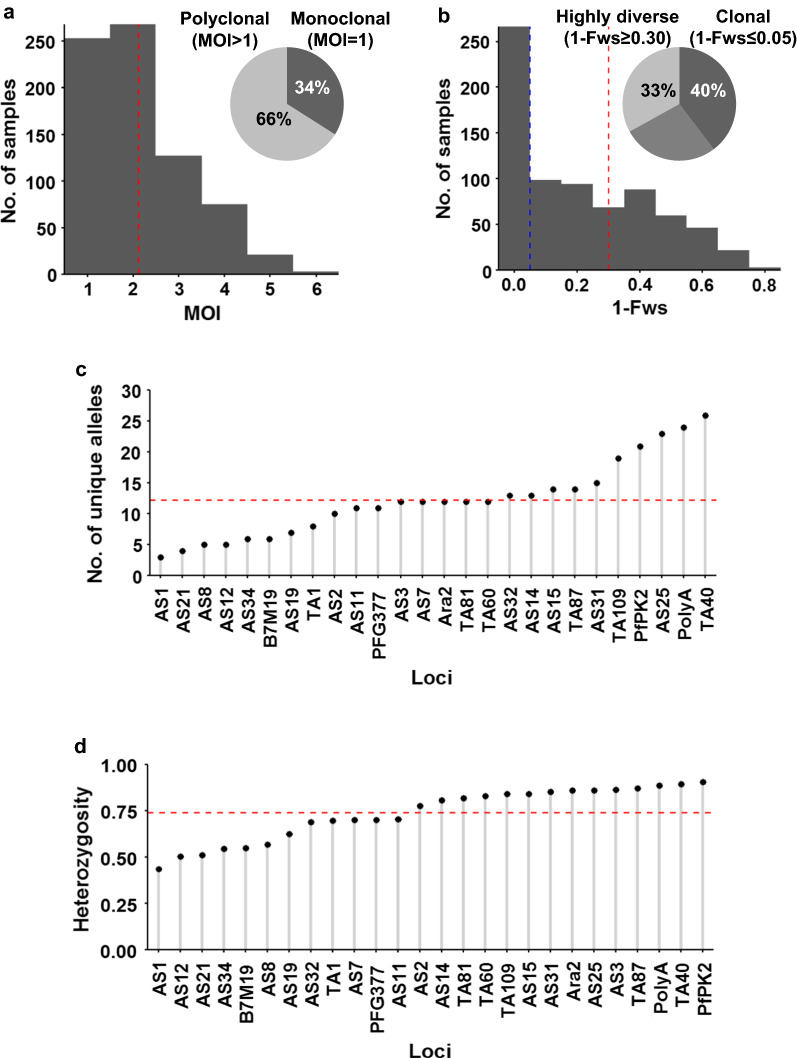
Within-host and population level diversity of *P. falciparum* parasites in the Vhembe District. **a** Multiplicity of infection (MOI) measured as the second highest number of alleles detected at any of the 26 loci. The dashed red line indicates the mean MOI = 2.13. The inserted pie chart shows proportion of single (34%) to multiple (66%) infections. **b** Within-host diversity index (1-Fws). 1-Fws value shows outbreeding and a value of 0 indicates a perfect clone. The dashed blue line indicates the cut-off of 1-Fws ≤ 0.05 which represents samples with clonal infections. The dashed red line indicates the cut-off of 1-Fws ≥ 0.30 which represents samples with high genetic diversity. The inserted pie chart shows proportion of clonal (40%) to highly diverse (33%) infections. **c** Distribution of number of unique alleles (allelic richness) detected in 26 microsatellite loci. The dashed red line indicates the mean = 12.2. **d** Population level genetic diversity measured as the distribution of heterozygosity in 26 microsatellite loci. The dashed red line indicates the mean heterozygosity = 0.74

Of the 1153 genotyped samples, 65% of the samples (747/1153) had sufficient coverage at a minimum of 15 of the 26 microsatellite loci evaluated and were included in the final sample set for population genetics analysis. Overall, a high proportion (66%) of polyclonal infections was observed with a mean MOI = 2.13 in the genotyped samples (Fig. 2a). This indicates a moderate complexity of infection within individual samples and thus moderate to high within-host diversity in the parasite population. Mean MOI did not differ significantly (*P* = 0.73, ANOVA, n = 353 excluding unknowns) between male (mean MOI = 2.11) and female participants (mean MOI = 2.16). The different age groups also exhibited similar mean MOIs (from 2 to 2.17) which were not significantly different (*P* = 0.94, ANOVA, n = 353 excluding unknowns).

A significant positive relationship (Pearson’s r = 0.85 [95% CI 0.83–0.87], *P* < 0.001, t-test, n = 747) was seen between outbreeding (1-F_WS_) and MOI, which suggests that both metrics support the presence of within-host diversity. Similarly to the one third of samples appearing monoclonal observed, only 40% of samples had 1-F_WS_ < 0.05 (Fws ≥ 0.95, indicating effectively clonal infections, Fig. 2b). Mean outbreeding (1-F_WS_) was low at 0.22, with only 33% of samples with a stringent 1-Fws value of ≥ 0.30 (Fws ≤ 0.70), suggesting that these were the most highly diverse infections (Fig. 2b).

On a population level, the majority of the samples (99%, 742/747) had unique haplotypes thus indicating high levels of outcrossing and, therefore, high genotypic diversity in the parasite population. The high number of unique haplotypes implies underlying allelic richness which is indeed reflected in the high mean number of unique alleles (mean A of 12.2) with anywhere from 3 to 26 unique alleles detected across all 26 loci (Fig. 2c). This was supported by a moderate to high mean *He* of 0.74 (Fig. 2d) that indicates frequent recombination of different parasite clones. This suggests a larger *P. falciparum* population than that expected in a low transmission setting, but is consistent with the Vhembe District being the highest transmission setting in South Africa. Locus PfPK2 was the most diverse marker (Nei’s genetic diversity of 0.91) and contributed to the high genotypic diversity whereas locus Ara2 had the most evenly distributed alleles (0.86) and, therefore, contributed to the most genotypic evenness. Overall, the sample set had a moderate to high genotypic richness, evenness and diversity.

Additionally, low LD (standardized index of association, ISA = 0.08) was observed between alleles of the *P. falciparum* haplotypes (Table 1) and this was not due to a single locus. The observed ISA value fell outside of the re-sampled distribution expected under no linkage when compared to histograms showing results of 10 000 permutations. The Monte Carlo method was used to test the significance of LD in the complete data set and alleles of monoclonal infections (n = 253) were linked across loci with *P* = 0.0001. For all infections including polyclonal infection, LD was also low at 0.138 but significant (*P* = 0.0001, n = 747). This was emphasized by a small proportion (0.26%, 221/85,078) of pairwise infections in the sample set being highly related (IBS ≥ 0.5). The significantly low LD therefore indicates high recombination of distinct parasite clones which supports the moderate to high within-host diversity observed in Vhembe, and is consistent with the presence of some degree of local transmission.

**Table 1:** Multi-locus linkage disequilibrium in *P. falciparum* populations of Vhembe district

Population	All infections		Single clones	
	n	ISA (*P* value)	n	ISA (*P* value)
Mutale	329	0.14 (0.0001)	111	0.03 (0.2633)
Unknown	393	0.14 (0.0001)	134	0.12 (0.0001)
Levubu/Shingwedzi	2	NA	1	NA
Thohoyandou	4	0.08 (0.0319)	3	-0.11 (1)
Nzhelele/Tshipise	17	0.14 (0.0001)	4	0.05 (0.3665)
Elim	2	NA	0	NA
TOTAL	747	0.138 (0.0001)	253	0.08 (0.0001)

n = number of isolates; ISA = standardised index of association; NA = not applicable

The Monte Carlo method was used to test the significance of LD

### Parasites are fragmented based on their level of within-host diversity

**Fig. 3:**
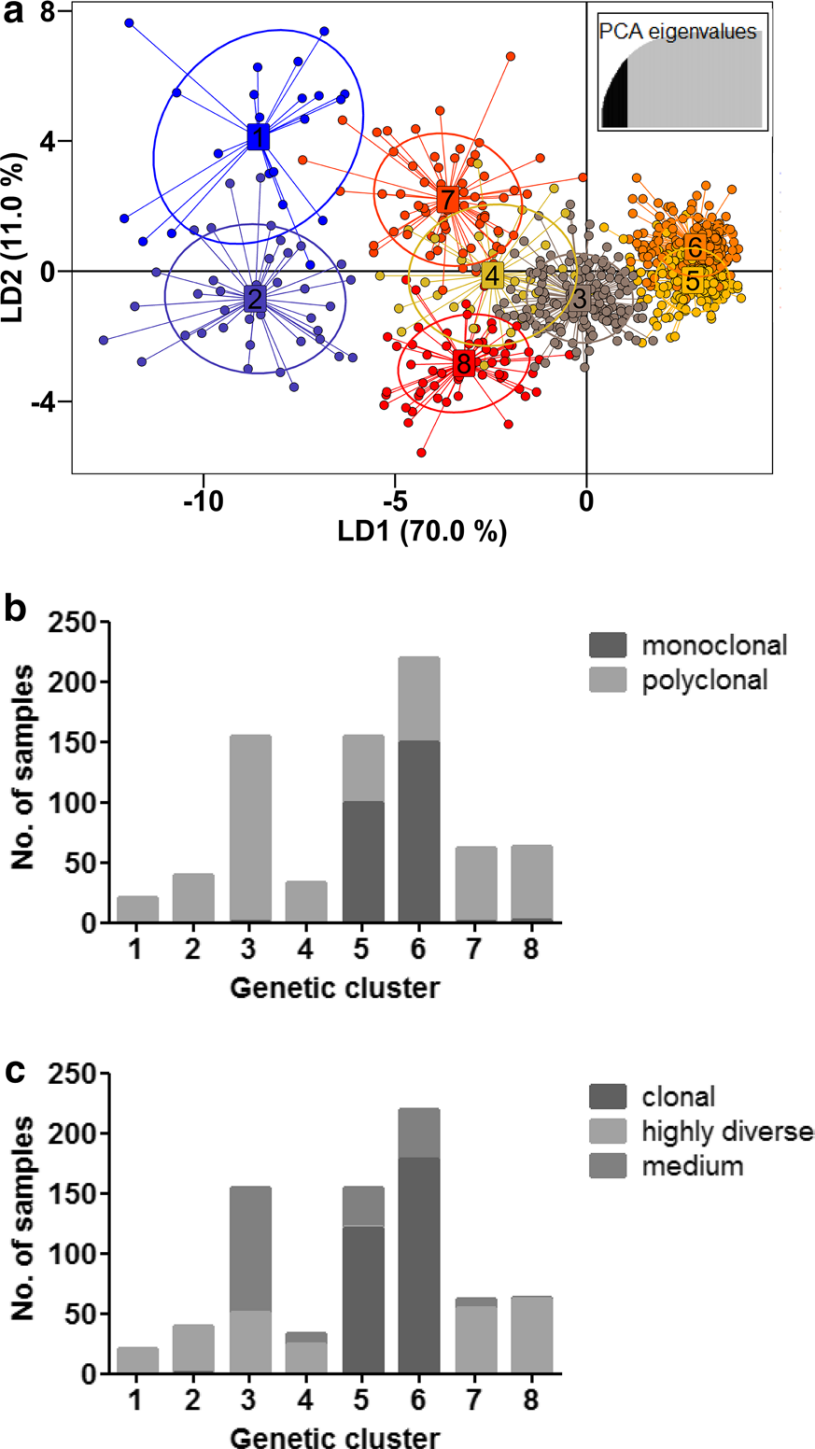
Fragmentation of parasites based on inferred genetic clusters. **a** Scatterplot of the discriminant analysis of principal components (DAPC) based on discrimination of the 8 inferred genetic clusters of *P. falciparum* populations. Samples whose source of infection is unknown are included. Individual multi-locus genotypes appear as dots. Colours and lines represent population membership. Analysis is based on retention of 50 principal components (top right insert). **b** Proportion of monoclonal (MOI = 1) to polyclonal (MOI > 1) infections in the fragmented genetic clusters. **c** Proportion of clonal (1-Fws ≤ 0.05) to highly diverse (1-Fws ≥ 0.30) infections. Medium represents infections with 1-Fws > 0.05 but < 0.30

To further evaluate the genetic relatedness between the parasite genotypes, k-means clustering was employed based on individual multi-locus genotype discrimination. Eight genetic clusters were identified in the parasite population with the parasite populations in genetic clusters 1, 2, 4, 7 and 8 observed using DAPC (Fig. 3a) separated by linear discriminant 1 (LD1) from parasites in clusters 5 and 6. Linear discriminant 2 (LD2) further separated clusters 2, 4 and 8 from clusters 1 and 7. The proportion of correct assignment of the haplotypes to each inferred cluster ranged between 0.95 and 1. The DAPC analysis could be performed with missing data in place with missing data which was randomly distributed in the data set basically replaced by the mean allele frequency in the case of multi-locus genotype (MLG) calculations. MLGs were generated from all major and minor allele data. Although all samples with any missing genotypes/data were included, loci PfPK2 and TA1 in which the majority of 83% and 90% of data was missing, respectively, were discarded in the DAPC analysis. Interestingly, clusters 5 and 6 contained the majority (98%, 249/253) of monoclonal (MOI = 1) infections (Fig. 3b). This was consistent with the mixture of mostly clonal (1-Fws < 0.05) and less diverse (medium, 1-Fws > 0.05 but < 0.30) infections in clusters 5 and 6 (Fig. 3c). Clusters 1 and 2, and to a lesser extent cluster 7, appeared fragmented and contained only highly diverse infections (1-Fws > 0.30, Fig. 3c). This indicates that although the majority of the parasite population is indeed structured, the most highly diverse infections result in fragmentation in the population. The moderate genetic diversity and MOI, and relatively pronounced population structure are all indicative of constant transmission at overall relatively moderate levels.

### No geospatial correlation exists to explain genetic diversity

**Fig. 4:**
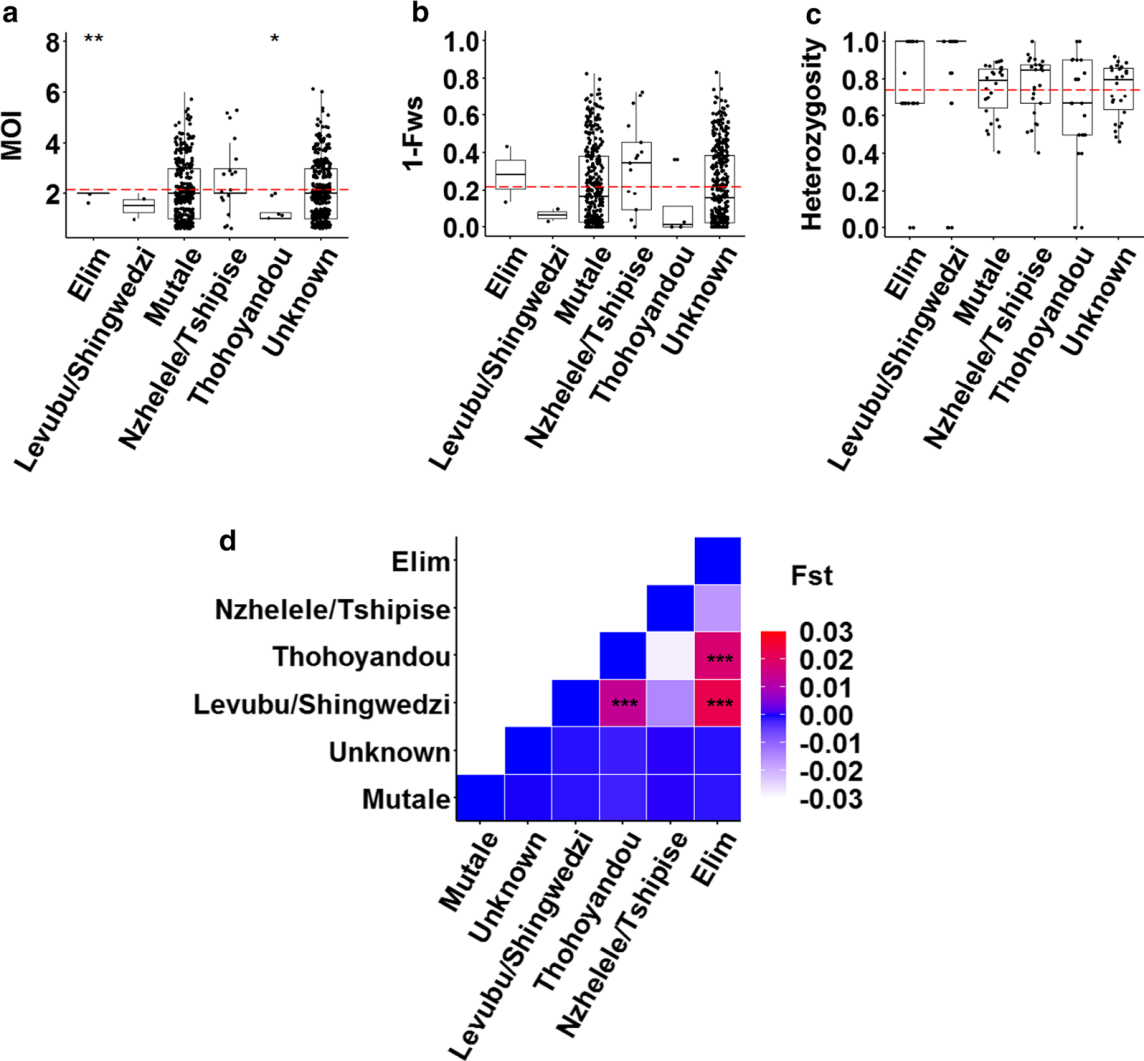
Geospatial variation of within-host and population level genetic diversity of *P. falciparum* parasites in Vhembe. **a** Multiplicity of infection (MOI) measured as the second highest number of alleles detected at any of the 26 loci across the different source health districts in the Vhembe District. The dashed red line indicates the mean MOI = 2.13. **b** Within-host diversity index (outbreeding, 1-F_WS_) measured across the different source health districts. The dashed red line indicates the mean 1-F_WS_ = 0.22. **c** Population level genetic diversity measured as the distribution of heterozygosity in 26 microsatellite loci across the different source health districts. The dashed red line indicates the mean heterozygosity = 0.74. Pairwise *P* values (t-test) are indicated in all plots where, not significant: *P* > 0.05; *: *P ≤* 0.05; **: *P ≤* 0.01; ***: *P ≤* 0.001; ****: *P* ≤ 0.0001. **d** Heatmap showing matrix of between source health district pairwise F_ST_. The range of F_ST_ values for the pairwise comparisons is shown in the legend and significance was tested using the Monte Carlo method

Overall, the levels of within-host and population level diversity were not influenced by where infections were reported since the mean MOI (global ANOVA *P* = 0.36, n = 747, Fig. 4a); the level of outbreeding (global ANOVA, *P* = 0.27, n = 747, Fig. 4b) and heterozygosity (global ANOVA *P* = 0.23, n = 747, Fig. 4c) between the different source health districts did not significantly differ. Only differences in the MOI from Elim and Thohoyandou were observed compared to the overall mean MOI (pairwise t-test, n = 747, *P ≤* 0.01 and *P* ≤ 0.05, respectively), which may be due to small sample size from these sites. The different source districts also did not contribute to the genetic clusters observed from the DAPC analysis, as infections from the different areas were represented/distributed in the eight different inferred genetic clusters, implying free gene flow and possible parasite mixing between these sites. This was confirmed by the very low F_ST_ values (Fig. 4d), with only the Elim and Levubu/Shingwedzi districts sharing significant F_ST_ with two other districts each, implying some degree of differentiation but again associated with small sample size from these areas. A Hendrick’s G_ST_ at − 0.0394 and Jost’s D at − 0.0122 supports general parasite mixing for the complete Vhembe district. This lack of separation based on geospatial data agrees with patient demographic and travel history information, with 99% of the infections locally acquired of which 95% are within the Mutale health district. This also correlated to residential status, with 93% of the individuals residing in Mutale. Additionally, 1-IBS analysis showed that infections were genetically connected within and between source areas, with highly related infections significantly higher (*P* < 0.0001, ANOVA, n = 221) within source areas (mean 1-IBS = 0.76 ± 0.022) compared to between source areas (mean 1-IBS = 0.56 ± 0.007) which confirms some level of local transmission.

### Transmission dynamics was not temporally influenced

**Fig. 5:**
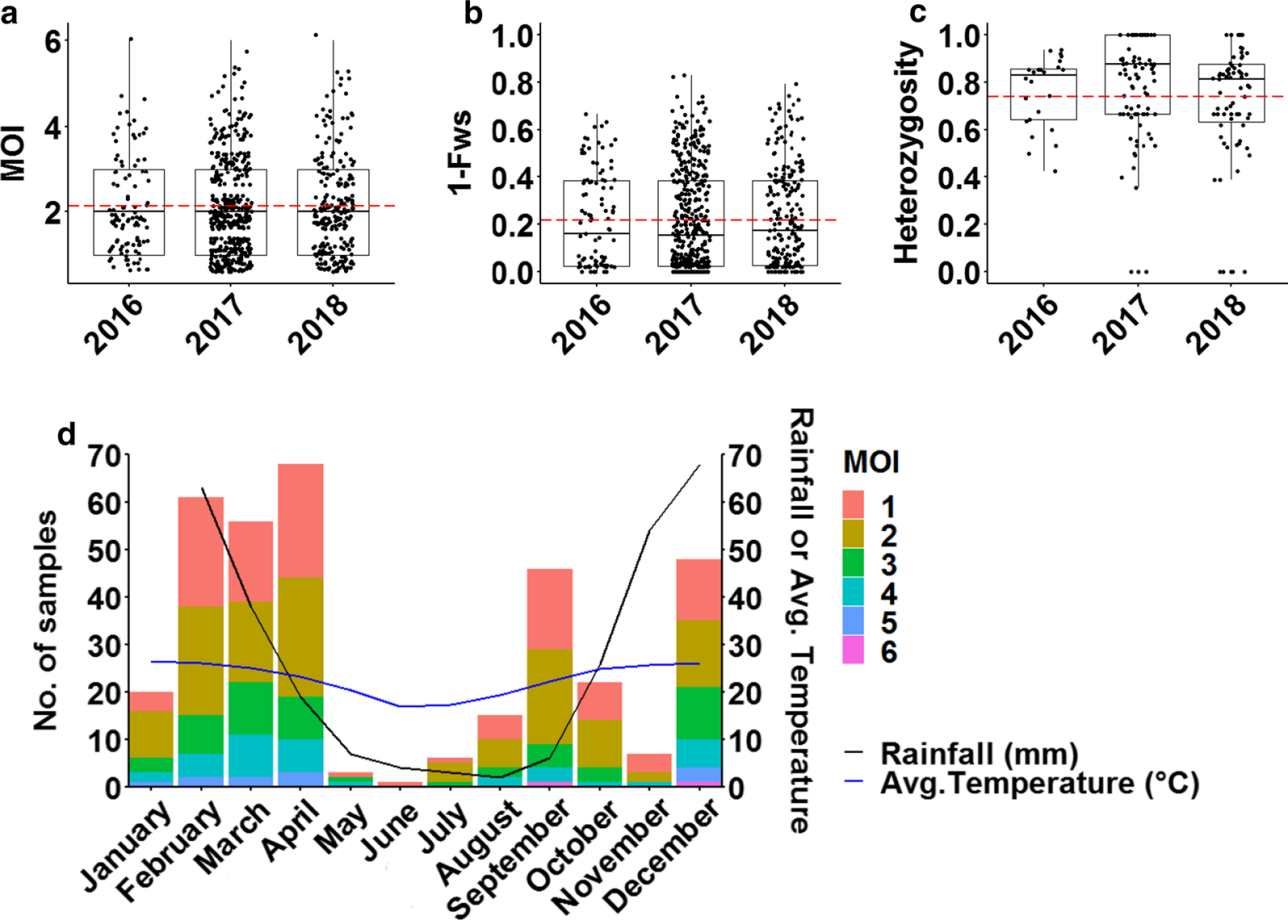
Temporal variation of within-host and population level genetic diversity of *P. falciparum* parasites in Vhembe. **a** Multiplicity of infection (MOI) measured as the second highest number of alleles detected at any of the 26 loci across the different years of transmission. The dashed red line indicates the mean MOI = 2.13. **b** Within-host diversity index (outbreeding, 1-F_WS_) measured across the different years. The dashed red line indicates the mean 1-Fws = 0.22. **c** Population level genetic diversity measured as the distribution of heterozygosity in 26 microsatellite loci across the different years from when the samples were collected. The dashed red line indicates the mean heterozygosity = 0.74. Pairwise *P* values (t-test) are indicated in all plots where, not significant: *P* > 0.05; *: *P ≤* 0.05; **: *P ≤* 0.01; ***: *P ≤* 0.001; ****: *P* ≤ 0.0001 **d** MOI distribution of collated samples throughout all the months of the year against different rainfall (black line) and temperature (blue line) patterns

The level of genetic diversity did not differ between the years of transmission as demonstrated by global ANOVA *P* values (n = 747) for MOI (Fig. 5a), outbreeding (Fig. 5b) and heterozygosity (Fig. 5c) of 0.90, 0.77 and 0.07, respectively, providing no evidence for differing transmission intensity during that period. No genetic differentiation (F_ST_ range − 0.00005 to 0.0003) between parasite populations from the different years was observed, which suggests that the malaria outbreak experienced in 2017 was not due to an introduction of a completely distinct/new parasite population to Vhembe.

Stratification of MOI distribution by transmission season (month of infection) (Fig. 5d) showed that infections were persistently complex throughout the year. In spite of an approximately 15-fold reduction in the number of cases and notable decrease in rainfall levels between the wet (high transmission) and dry (low transmission) seasons (Fig. 5d), mean MOI was also not significantly different (*P* = 0.42, Bonferroni *P* adjustment method) between the two seasons (wet season mean MOI = 2.14 ± 0.056, and mean MOI = 1.96 ± 0.178 in the dry season). Infections were similarly complex (mean MOI ± SE = 2.15 ± 0.075 and 2.14 ± 0.101; *P* = 1, Bonferroni *P* adjustment method) between infections from sprayed or unsprayed households suggesting the maintenance of complex infections in spite of vector control implementation during wet seasons, suggesting possible co-transmission of strains. These data may indicate that the mean MOI may be seasonally stable thus reflecting the impact of continued residual transmission on the complexity of infections in the Vhembe District. Out of the 220 patients who knew which insecticides were sprayed in their households, 53% (116/220), 44% (97/220) and 3% (7/220) reported use of DDT, Fendona® and K-Othrine®, respectively.

## Discussion

The impact of malaria transmission intensity on *P. falciparum* parasite genetic diversity is only now being clarified in South Africa. By using multilocus genotyping, this is the first study that shows that continued residual malaria transmission in a malaria hotspot within South Africa is associated with parasite diversity.

The results generated in this study are a useful addition to the growing resource of *P. falciparum* genetic data in the southern African E8 region which will facilitate more detailed evaluations of cross-border parasite spread in future studies. The genetic data may also be useful to control programmes with decision making for malaria elimination as it can be used as a reference point for the assessment of the effectiveness of on-going interventions over time, selection and/or targeting of interventions. Furthermore, the genetic data may be used for the identification of imported cases and/or outbreaks, as well as monitoring for the potential spread of anti-malarial drug resistance and potentially revealing infection patterns.

Although South Africa is overall a low transmission setting, the Vhembe District is a transmission hotspot and here it is shown that the parasite population within this hotspot behaves genetically like those typically seen in high transmission settings (based on incidence data) such as Guinea, Mali and The Gambia [16, 40–42] - the *P. falciparum* parasite population in the Vhembe District is complex and diverse. This is typically associated with high levels of gene flow between areas of different transmission intensities that serve to compound allelic richness by introducing new alleles into the population, thereby increasing the level of heterozygosity in the parasite population [9, 25]. Although the Vhembe district is located along the border with Mozambique and Zimbabwe, very limited evidence exists for imported malaria cases and rather, the high level of heterozygosity observed in this study implies localised diversity in a transmission hotspot, where relatively higher transmission occurs compared to other areas in the country. This is supported by a marked level of gene flow and parasite mixing between parasite populations from the different source areas within the district, resulting in a low but significant LD, with frequent and random association between alleles and a panmictic parasite population [43]. Geospatial and temporal variances had little effect on within-host and population diversity in the Vhembe District suggesting that genetic diversity was stable over space and time. The fact that mean MOI remained seasonally stable may be a reflection of the contribution of complex infections to continued residual transmission in the Vhembe District.

A major limitation of this study was that a large number of samples were classified as ‘unknown’ and could not be linked to their district of origin, thereby leaving the geospatial analyses to a certain extent underpowered in most districts. Additionally, the majority of the samples coming from the Mutale district may have been influenced by a biased sampling plan from the onset based on the fact that samples were collected from a few selected clinics in a known hotspot area based on case incidence data. It was, therefore, not possible to get genetic representation of parasites from the other source districts and those seeded through importation from neighbouring high transmission settings reported in the province if any and what their contribution to the local transmission dynamics is.

Hotspot areas in other low transmission settings, such as Malaysia have demonstrated opposite trends where parasite complexity and heterozygosity are low, and LD is high using both microsatellite markers and merozoite surface protein variants for genotyping [21, 44]. In Madagascar, however, high levels of genotypic diversity and a high proportion of polyclonal infections were associated with a transmission hotspot using single nucleotide polymorphism genotyping [45]. These differences may have been due to differences in technology platforms used for genotyping in comparison to this study or due to the challenge of non-standardisation of study designs in molecular epidemiology studies. Additionally, since “low” in Africa is very different than “low” in Asia (which is generally much lower based on for example entomological inoculation rates and parasite prevalence rates) it could have also been due to differences in underlying epidemiology of parasite transmission between Malaysia and Madagascar. In other low transmission areas of southern Africa [9, 25, 26], where a similar panel of microsatellite markers was used, the level of genetic diversity was as high as that in the Vhembe District. However, in Eswatini and in the KZN province of South Africa, the high levels of within-host and population diversity and lack of parasite population structure could be explained by high levels of importation from neighbouring high transmission areas. On the other hand, in north-eastern Namibia, while infections were genetically diverse, transmission was mostly as a result of locally acquired infections and fine-scale parasite fragmentation based on the geographic origin of parasites was observed [26]. Detectable genetic clusters of different genotypes mean if studies are strategically designed and patient parasite genetic and demographic data accurately linked in all remaining endemic areas in South Africa and neighbouring endemic countries, particularly at the border areas, parasites from different countries can easily be detected or traced back to their origins. This information can then be used to make better decisions on a national level and as a regional block on what interventions should be put in place, and concentrated in which areas. Therefore, regional comparison of parasite genotypes will be informative to understand parasite mixing in the context of malaria transmission.

Some clones in an infection may exist at lower proportions than others due to either competitive suppression by other genotypes or possibly host specific selection due to immunity or receptor polymorphisms. In *Plasmodium chabaudi*, minor clones in mixed/multiple infections may produce as many, or more, oocysts than they would have as a single clone infection [46], highlighting that competitive stress may increase transmission of certain clones. Multiple distinct parasite clones are implicated in high gametocyte production and emergence of highly virulent and drug resistant parasite strains due to intense within-host competition [47–50]. While the relationship between parasite density and MOI is complex, and may not be enough to explain possible within-host competition of parasite clones in individual infections in the Vhembe District, a detailed, longitudinal study of the contribution of specific clones to parasite transmissibility and virulence would therefore be important.

## Conclusion

In this study, the impact of continued malaria transmission intensity on *P. falciparum* genetic diversity in the Vhembe District is demonstrated. The *P. falciparum* population is moderate to highly diverse and genetically complex, which is key and advantageous to the parasite’s evolution and survival. This data could be informative as a reference point in evaluating the efficacy of strategic control interventions over time, aimed at eliminating residual malaria transmission in malaria transmission ‘hotspots’ in South Africa. Furthermore, this data can be used to identify imported cases and/or outbreaks, as well as monitor for the potential spread of antimalarial drug resistance. Linking data on *P. falciparum* genetic diversity from Vhembe/South Africa to that from neighbouring sub-Saharan countries in the E8 regional initiative would need to be investigated as the transmission dynamics in the region is not fully understood.

## Data Availability

The datasets supporting the conclusions of this article are available from the corresponding authors on reasonable request.

## Abbreviations

DAPC: Discriminant analysis of principal components
IRS: Indoor residual spraying
KZN: Kwa-Zulu Natal
LD: Linkage disequilibrium
LMC: Limpopo Malaria Case
MOI: Multiplicity of infection
PCA: Principal component analysis
RDT: Rapid diagnostic tests
